# Transcriptome profiling of eight *Zea mays* lines identifies genes responsible for the resistance to *Fusarium verticillioides*

**DOI:** 10.1186/s12870-024-05697-y

**Published:** 2024-11-21

**Authors:** Thi Nhien Tran, Alessandra Lanubile, Adriano Marocco, Mario Enrico Pè, Matteo Dell’Acqua, Mara Miculan

**Affiliations:** 1 Center of Plant Sciences, Scuola Superiore Sant’Anna, Pisa, 56127 Italy; 2 Department of Sustainable Crop Production, Università Cattolica del Sacro Cuore, Piacenza, 29122 Italy; 3 Present Address: Biological and Environmental Science and Engineering Division (BESE), King Abdullah University of Science and Technology, Thuwal, 23955 Saudi Arabia; 4 Present Address: Cuu Long Delta Rice Research Institute, Tan Thanh Commune, Can Tho City, Thoi Lai District 94700 Vietnam

**Keywords:** *Fusarium verticillioides*, MAGIC population, RNA sequencing, Rolled Towel Assay, WGCNA, *Zea mays*

## Abstract

**Background:**

The cultivation of maize (*Zea mays* L.), one of the most important crops worldwide for food, feed, biofuels, and industrial applications, faces significant constraints due to *Fusarium verticillioides*, a fungus responsible for severe diseases including seedling blights, stalk rot, and ear rot. Its impact is worsened by the fact that chemical and agronomic measures used to control the infection are often inefficient. Hence, genetic resistance is considered the most reliable resource to reduce the damage. This study aims to elucidate the genetic basis of *F. verticillioides* resistance in maize.

**Results:**

Young seedlings of eight divergent maize lines, founders of the MAGIC population, were artificially inoculated with a *F. verticillioides* strain. Phenotypic analysis and transcriptome sequencing of both control and treated samples identified several hundred differentially expressed genes enriched in metabolic processes associated with terpene synthesis. A WGCNA further refined the pool of genes with potential implications in disease response and found a limited set of hub genes, encoding bZIP and MYB transcription factors, or involved in carbohydrate metabolism, solute transport processes, calcium signaling, and lipid pathways. Finally, additional gene resources were provided by combining transcriptomic data with previous QTL mapping, thereby shedding light on the molecular mechanisms in the maize-*F. verticillioides* interaction.

**Conclusions:**

The transcriptome profiling of eight divergent MAGIC maize founder lines with contrasting levels of *Fusarium verticillioides* resistance combined with phenotypic analysis, clarifies the molecular mechanisms underlying the maize-*F. verticillioides* interaction.

**Supplementary Information:**

The online version contains supplementary material available at 10.1186/s12870-024-05697-y.

## Background

Maize (*Zea mays* L.) is among the most important crops worldwide for food, feed, biofuels, and industrial applications. With more than one billion tons harvested every year, it ranks second globally for total production [1]. Across all its cultivation range, maize faces significant constraints due to the presence of pathogens affecting the quality and quantity of its production. Fungal diseases may have a devastating impact on maize cultivation, depending on many factors, including environmental conditions, susceptibility of maize varieties, and agronomic practices [2].

*Fusarium verticillioides* (Sacc.) Nirenberg is considered the most widespread fungal pathogen of maize worldwide [3]; its infection may cause considerable yield losses and a drastic reduction of grain quality. The fungus can colonize several maize tissues at different environmental stages causing manifold diseases, including Fusarium seedling rot (FSR), Fusarium ear rot (FER) and Fusarium stalk rot (FStR) [4, 5]. Moreover, the pathogen may impact human and animal health by feeding on contaminated maize, since the fungus synthesizes a wide range of mycotoxins, primarily fumonisins, that are cancerogenic [6, 7]. Current chemical and agronomic measures to control *Fusarium verticillioides* infection are largely inefficient [7] and call for the development of durable genetic resistance.

The genetic basis of maize resistance to *F. verticillioides* is not yet fully elucidated, and most research has been centered on the host resistance to FER. The phenotypic evaluation of this disease entails field trials employing different inoculation techniques [8, 9]. However, field assessments are time-consuming and must be repeated across several environments for different years [8, 10–13]. Moreover, disease evaluation is profitable only when environmental conditions are suitable for fungal growth and spread. In contrast, at the seedling stage, the estimation of genetic resistance to FSR may profit from laboratory assays by which seed infection with *F. verticillioides* occurs under controlled inoculation conditions and spore concentrations [14–17]. Even though FER and FSR represent two distinct diseases and concern different growth stages of the plant, germplasm screening for resistance at seedling stage may constitute a decisive strategy to hinder fungal disease and fumonisin contamination. In this regard, previous works used different maize panels and populations to study FSR by combining Genome-Wide Association Studies (GWAS) and transcriptional analyses [14–17]. RNA sequencing (RNA-seq) approach is a powerful method to identify transcriptional clues reporting molecular pathways underpinning phenotypes. For this reason, transcriptional studies have been applied to study expression patterns in response to fungal attacks in maize, reporting complex networks of genes that may enhance resistance [9, 18]. Using a combination of these methods, candidate genes and genomic loci with a potential role in disease resistance, albeit partial, were described. For instance, by incorporating GWAS and QTL mapping, it was possible to identify eight QTL and 43 genes associated with FSR resistance [14]. In the study by Septiani et al. [15] eight candidate genes within the three identified FSR QTL were pointed out using a Multi-parent Advanced Generation Inter Crosses (MAGIC) population that offers great potential for improving breeding populations as well as for high-resolution trait mapping [15, 19]. A complete understanding of the molecular mechanisms underlying the infection process and the corresponding resistance pathways is crucial for developing innovative breeding programs incorporating disease resistance to cultivated maize.

Weighted gene co-expression network analysis (WGCNA) is a popular systems biology tool used for studying gene correlations, identifying modules highly associated with phenotypes and detecting hub genes within these modules [20, 21]. Based on the co-expression relationships, genes with similar expression are grouped into the same module, thus suggesting that these genes may have similar functions or possibly have common biological regulatory roles. This method has been successfully applied in various genomic studies that have been used to identify hub genes, and to find the relationships between gene expression data and relevant plant phenotypes, including resistance to fungal diseases [22–26].

The aim of this work was to investigate early differences in transcriptional regulation after *F. verticillioides* inoculation using eight diverging maize lines that are the founders of a MAGIC population [19]. The eight founder lines encompass most of the genetic and geographic diversity of the essential public lines historically and currently used in maize breeding, representing the major maize breeding groups/subgroups, thus holding high agronomic importance [27]. Leveraging the diversity existing in the dataset, we produced a transcriptomic dataset aimed at addressing three biological questions associated with FSR in maize: (1) which transcriptional perturbations are induced by the *F. verticillioides* inoculation in different maize lines; (2) which transcriptional perturbations are induced by *F. verticillioides* inoculation in maize as a whole; (3) which genes are specifically responsible for the resistance to *F. verticillioides* in maize. To answer these questions, we used a combination of: differential expression analysis, WGCNA, and gene ontology. Since resistance to pathogens is a complex polygenic trait, we hypothesize that the disease response activates specific pathways rather than just a few genes. In fact, we found several hundred differentially expressed genes strongly enriched in metabolic processes associated with terpene synthesis. WGCNA further refined the pool of genes with potential implications in disease response and identified a limited set of hub genes such as those encoding bZIP and MYB transcription factors, or involved in carbohydrate metabolism, solute transport processes, calcium signaling and lipid pathways. Finally, combining transcriptomic data with previous QTL mapping, additional gene resources that could be used to develop *F. verticillioides* resistant genotypes were provided.

## Methods

### Plant material and inoculation bioassay

The eight genetically diverse maize inbred lines (A632, B73, B96, F7, H99, HP301, Mo17 and W153R), founders of the MAGIC maize population [19] and used in this study, were obtained from Scuola Superiore Sant’Anna, Pisa, Italy, and maintained by sibling at the Department of Sustainable Crop Production, Università Cattolica del Sacro Cuore, Piacenza, Italy.

Mature kernels of each inbred line were artificially inoculated using the Rolled Towel Assay (RTA) phenotyping method [15]. One RTA is considered a biological replicate. One hundred and twenty seeds with similar size and without visible damage were chosen for each MAGIC Maize (MM) founder line, sixty to be used for *F. verticillioides* inoculation treatment (20 seeds each replicate, 3 biological replicates), and sixty for the control condition (20 seeds each replicate, 3 biological replicates). Before running the experiment, seeds were sterilized as previously described [15, 17, 28]. For each RTA, twenty seeds were placed on two moistened towels of germinating paper (Anchor paper, Saint Paul, MN, USA), inoculated with 100 µl of 1 × 10^6^ conidial suspension of *F. verticillioides* ITEM10027 (MPVP 294) and covered with a third towel. The strain was isolated from maize in South Tuscany, Italy, by the Department of Sustainable Crop Production, Università Cattolica del Sacro Cuore, Piacenza Italy, and stored in their fungal collection, and also in the Institute of Sciences and Food production, National Research Council of Bari, Italy (http://server.ispa.cnr.it/ITEM/Collection). In control, RTAs seeds were not inoculated with the conidial suspension of *F. verticillioides*. For both conditions, the towels were then rolled-up, placed vertically in a bucket, and put in transparent plastic bags to keep treated and control RTAs separated to avoid cross-contamination. In total, six RTAs, three biological replicates for treatment and three biological replicates for control conditions, were carried out for each MM founder line, for a total of 48 RTAs.

To quantify the plant response to the disease and detect the levels of *F. verticillioides*, by real-time reverse transcription-quantitative PCR (RT-qPCR), RTAs of lines H99 and Mo17 were incubated at 25 °C in the dark for 48, 72, 120 and 168 hpi after the inoculation step. Based on the expression trend of fungal and maize genes during the seven-day time-course in the two lines, only the 72 hpi time-point was selected for RNA-seq analysis across all MM founder lines. At the end of the incubation period, the phenotypic evaluation of FSR was performed using a scale from 1 to 5, as previously reported [15, 17, 28].

Maize scutella tissues were dissected from maize seedling samples of each parental line, immediately frozen in liquid nitrogen and stored at -80 °C until both RT-qPCR and RNA-Seq analysis were carried out.

### RNA isolation

The twenty seeds from each RTA were pooled and considered as one single biological replicate. Total RNA extraction and purification were carried out according to Lanubile et al. [29]. RNA sample concentration was assessed by fluorometric assay (Qubit, Thermo Fisher Scientific Inc. Waltham, MA, USA), and quality was checked through A260/280 and A260/230 ratios obtained with a Nanophotometer NP80 (IMPLEN, München, Germany) and by agarose gel electrophoresis.

### Real-time RT-qPCR expression analysis of *β-tubulin* and *pathogenesis-related maize 3* genes

As mentioned above, real-time reverse transcription-quantitative PCR experiments were performed on scutella tissues collected at 48, 72, 96, 120 and 168 hpi for the lines H99 and Mo17 using the FluoCycleTM II SYBR Green master mix (EuroClone S.p.a., Milan, Italy) and the CFX-96 device (Bio-Rad, Hercules, CA, USA). Scutella tissues were examined instead of the entire seed given their strategic role in mediating biotic stress protection as rich sources of nutrients and defense chemicals [30]. Moreover, previous research by Septiani et al. [15] demonstrated that the founder lines H99 and Mo17 exhibited highly resistant and highly susceptible phenotypes to *F. verticillioides*, respectively.

One µg of total RNA was taken for cDNA synthesis using the High-Capacity cDNA Reverse Transcription Kit (Thermo Fisher Scientific). Twenty ng of single strand cDNA determined by fluorometric assay (Qubit, Thermo Fisher Scientific) were used for RT-qPCR.

The real-time qPCR assay was used to quantify the growth of *F. verticillioides*, detecting the copy number of the fungal house-keeping gene *β-tubulin* transcripts with the following primer pairs: 5′-ACA TCC AGA CAG CCC TTT GTG-3′ (forward) and 5′-AGT TTC CGA TGA AGG TCG AAG A-3′ (reverse), and with the following thermal cycling conditions: one initial step at 95 °C for 3 min followed by 35 amplification cycles (95 °C for 40 s, 56.7 °C for 40 s and 72 °C for 40 s), and finally 72 °C for 10 min, as previously reported [31]. The number of *β-tubulin* copies is related to the quantity in nanograms of cDNA obtained from maize scutella tissues and determined based on a linear regression equation according to the technical manual (Bio-Rad). To determine the fungal cDNA copy number, each sample of kernel cDNA (20 ng) was compared to a dilution standard curve obtained by serially diluting [1:1, 1:5, 1:5^2^, 1:5^3^, 1:5^4^, 1:5^5^] 20 ng of fungal cDNA from *F. verticillioides* isolate ITEM10027 in sterile water.

To quantify the plant response to the disease, the relative expression of the gene *pathogenesis-related maize 3* (*PRm3*) was performed, and the primer pairs 5′-GGC TCT ACG CCT ACG TCA AC-3′ (forward) and 5′-GAT GGA GAG GAG CAC CTT GA-3′ (reverse) were used, as previously reported [32]. Relative RT-qPCR was performed under the following conditions: 95 °C for 3 min and 40 cycles at 95 °C 15 s, 57 °C for 30 s, followed by a melting curve analysis [32]. Three technical replicates (within each biological replicate) were employed for each tested sample and template‐free negative controls. Relative quantification was normalized to the reference housekeeping gene *β‐actin* using the following primer pairs: 5′- ATG GTC AAG GCC GGT TTC G-3′ (forward) and 5′-TCA GGA TGC CTC TCT TGG CC-3′ (reverse) [32]. Fold changes (FC) values in gene expression were calculated using the 2^−ΔΔCt^ method [33] and calibrated on the control kernels.

### Library preparation and transcriptome sequencing

As disclosed above, for RNAseq analysis, only the time-point 72 hpi was considered for all MM founder lines. A total of cDNA 48 libraries (eight genotypes x two conditions x three biological replicates) were constructed following the manufacturer’s instructions of the Illumina TruSeq Stranded mRNA kit (Illumina, San Diego, CA), and then paired-end sequenced (2 × 150 bp) with the NovaSeq6000 platform (Illumina, San Diego, CA) at IGA-Tech (Udine, Italy).

### Differential gene expression analysis

Raw reads were demultiplexed, processed for adapter removal and trimmed for quality by Cutadapt v1.11 [34] based on a Phred quality score (bases retained if Phred score is greater than 30). The quality of trimmed reads was assessed by FastQC v0.11.5 (Andrews, 2010). High-quality reads were mapped against the *Zea mays* reference genome assembly B73 (v4.44) with STAR read aligner V.2.7.3a [35] using the dual-mode approach. Raw gene counts were quantified with the parameter *quantMode GeneCounts* within the alignment step by STAR read aligner. An empirical Bayes model implemented in the R package *edgeR* [36] was used to moderate the degree of overdispersion (mean-variance relationship) across genes, obtaining the Counts Per Million (CPM) normalization. The dispersion of each gene was estimated through the function *estimateDisp()* in *edgeR*. The formula *model.matrix()* was run to construct the design matrix to be used in the generalized linear model (GLM) approach. Among the many equivalent ways to define the design matrix, a coefficient for expression level was chosen for each group, where a group corresponds to the combination of line plus treatment: for example, for line A632, we defined a group level for the control (*A632.control*) and a group level for the treated condition (*A632.treated*). The intercept column was not included in the design matrix. Once dispersion estimations were obtained and negative bimodal GLM were fitted, a gene-wise quasi-likelihood (QL) F-tests (an exact test analogous to Fisher’s exact test adapted for overdispersed data), was used to assess the differential expression analysis (*glmQLFit()* function, *edgeR* package). We performed the analysis multiple times to find groups of differentially expressed genes able to answer the following biological questions: (1) effect of *Fusarium verticillioides* infection in *Zea mays* (*inoculation* vs. *control* across inbred lines); (2) effect of *F. verticillioides* infection within each line (*inoculation* vs. *control* within each line separately); (3) effect of *F. verticillioides* infection in *resistant* vs. *susceptible* lines. The contrasts applied in the *glmQLFit()* function are shown in the Methods S1. For the purposes of this work, we considered a gene as differentially expressed, if the adjusted *p-value* (Benjamini–Hochberg method) is below 0.05, regardless the fold-change.

### Weighted gene co-expression network analysis

Weighted gene co-expression network analysis (WGCNA) was performed using the R package WGCNA v1.63 [21, 37]. A signed network was constructed separately for control samples (*Control-Network*) and for treated samples (*Treated-Network*) following the step-by-step tutorial suggested from Langfelder and Horvath [21]. The adjacency matrix was calculated with a soft threshold power of 8 for both datasets, and modules were forced to contain at least 25 genes. Modules with correlation > 0.8 were merged. The correlation of a gene with all other genes in the network (i.e. Gene Connectivity), was obtained through the function *intramodularConnectivity()* from the adjacency matrix. Module preservation statistics was computed with the WGCNA package formula *modulePreservation()* setting the *Control-Network* as reference and the *Treated-Network* as test.

Statistical overrepresentation test analysis of the WGCNA modules was conducted through the Protein ANalysis THrough Evolutionary Relationships (PANTHER) Classification System [38, 39]. Only terms with FDR < 0.05 (Fisher exact test) were considered significative.

### Gene Ontology analysis

Gene Ontology (GO) and pathway enrichment of differentially expressed genes (DEG) were analyzed employing the online database PANTHER v17.0 (www.pantherdb.org) [38, 40, 41]. PANTHER is a publicly available, user-focused knowledgebase providing many functional annotation tools for investigators to understand the biological importance behind a long list of genes. The *P* value for the significance of the gene-enrichment term listed by PANTHER was obtained through over-representation analysis by Fisher’s exact test and the results were corrected for the FDR using the Benjamini–Hochberg method [38, 40, 41].

### Source of candidate genes

Transcriptomic data generated on the founder lines was used to guide the identification of candidate genes within QTL previously identified by Septiani et al. [15]. The 2-LOD confidence limit of the QTL for FSR resistance was considered for the search of candidate genes. The physical coordinates of the interval limits were determined on the *Zea mays* genome assembly B73 (v4.44). Genomic positions of the QTL were compared with the list of differentially expressed genes deriving from the three contrasts considered in this study.

## Results and discussion

### Highest molecular response to inoculation of *F. verticillioides* occurs at 72 h post inoculation

To identify genes associated with the resistance to *F. verticillioides*, we performed a transcriptome analysis of the eight founder lines of the MAGIC Maize (MM) population [19] along with the investigation of phenotypic variation induced by the inoculation. To select the relevant time-point to perform RNA-Seq analysis, we conducted an explorative time-course experiment of five time-points (from 48 to 168 h post-inoculation, hpi) on seedlings of the highly resistant and of the highly susceptible lines (H99 and Mo17, respectively) [15]. The Rolled Towel Assay (RTA) showed a different pattern of Fusarium Seedling Rot development in the two lines (Fig. S1A). In the highly resistant line H99, disease severity increased gradually, reaching the maximum value of 2.2 (minimum level = 1, maximum level = 5) at 168 hpi, indicating a reduced presence of *F. verticillioides.* Quite the opposite, in the highly susceptible line Mo17, the FSR value increased significantly after 72 hpi, reaching the maximum level of 4.8 at 96 hpi, and continued to exhibit a high severity incidence up to 168 hpi (4.4; Fig. S1A). These results were in line with those obtained through the fungal growth assay by the absolute quantification of the *F. verticillioides β-tubulin* housekeeping gene (Fig. S1B). In seedlings of the highly susceptible line Mo17, *β-tubulin* copy number (Copy N°) could be appreciated already at 72 hpi (Copy N° = 196.8), whereas only at 96 hpi in H99 (Copy N° = 384.3; Fig. S1B), confirming the delayed and slight fungal growth in the resistant line. Moreover, we quantified the expression of the maize *PRm3* gene to have an indication of the molecular defense response of maize to the fungus (Fig. S1C). Pathogenesis-related maize (PRm) proteins are products of defense genes and have been often considered as markers for the resistance to fungal pathogens. The contribution of PRms to kernel resistance was previously demonstrated in response to several mycotoxigenic fungi, including *F. verticillioides* [32, 42], *F. graminearum* [43] and *Aspergillus flavus* [32, 42, 44, 45]. As expected, the highly resistant line H99 showed a quicker response already at 48 hpi with a FC of 2.7 compared to the highly susceptible line Mo17, where *PRm3* reached the maximum level of expression at 72 hpi (FC = 6.3; Fig. S1C). In a previous work in 2016, Maschietto et al. [46] found higher expression of pathogenesis-related genes (including the same *PRm3* gene) and antioxidant enzymatic activities in mock-inoculated kernels of two maize resistant lines; in contrast, the susceptible genotypes activated defensive genes at increased levels only at the late timepoint (72 hpi) after *F. verticillioides* inoculation. This trend highlighted possible constitutive defense mechanisms present in the resistant background such as to allow the more rapid reaction observed in the H99 genotype in the first 48 hpi.

All together these results indicated that the main reactions of maize to pathogen attack took place in the first 72 hpi, and for this reason we considered 72 hpi as the most suitable time-point to investigate the transcriptomic variability among the MM founder lines following *F. verticillioides* inoculation.

### The Fusarium Seedling rot severity varies across the MAGIC maize parental lines

**Fig. 1:**
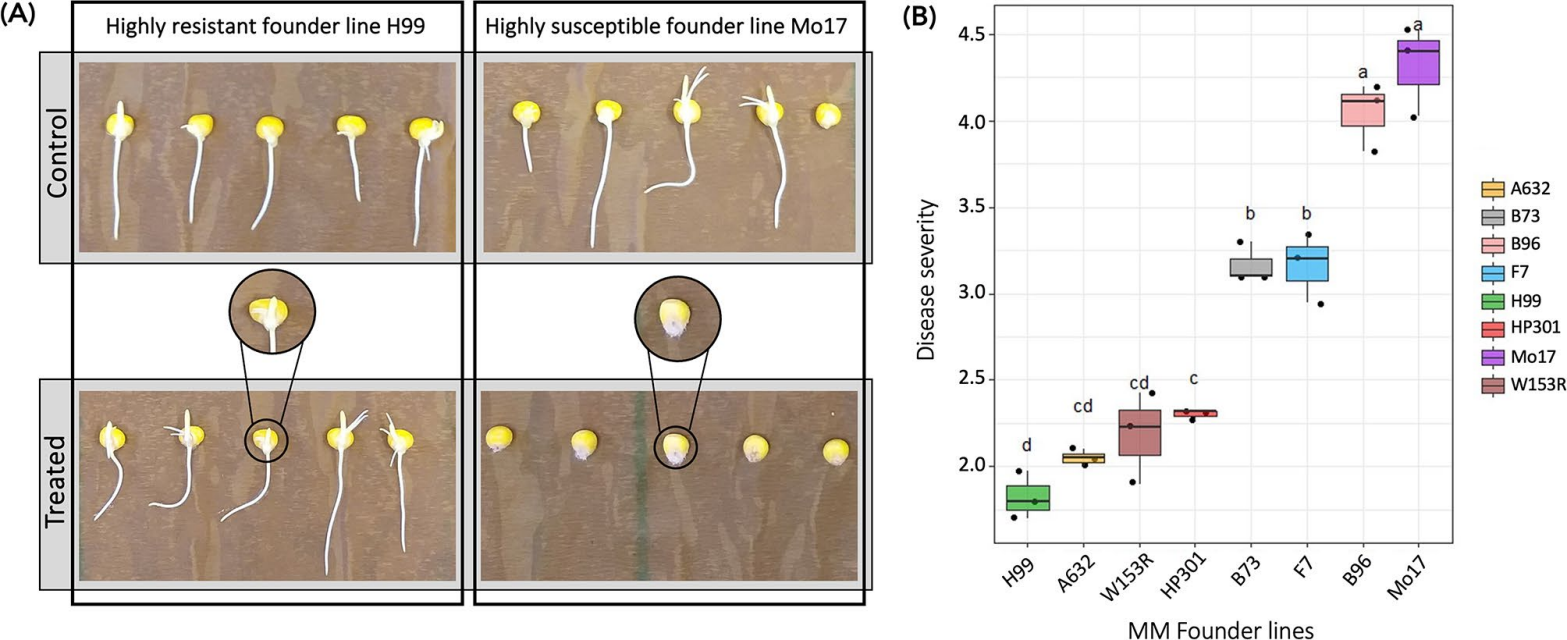
Fusarium Seedling Rot (FSR) evaluation in the eight MAGIC Maize (MM) founder lines at 72 hpi with *Fusarium verticillioides*. FSR disease severity in seedlings was evaluated assigning a score ranging from 1 to 5 as reported in Septiani et al. (2019), according to the level of infection: score 1 indicates minimum or no effects (resistant phenotype), while score 5 means that the seed is severely affected (susceptible phenotype). **(A)** Phenotypic variation in FSR severity among seedlings of the highly resistant H99 and the highly susceptible Mo17 founder lines. **(B)** FSR scores observed in the eight MM founder lines considering three biological replicates; letters on the box and whiskers plots are significantly different at *p* < 0.05 in the LSD test

We conducted a phenotypic evaluation of FSR on all eight founder lines at 72 hpi using RTAs and scoring the response to *F. verticillioides* inoculation. We considered two conditions: control (mock inoculation) and treated (inoculation with conidial suspension). The RTA screening showed a wide range of phenotypic variation for disease severity (Fig. 1), scored by visual evaluation from 1 (null) to 5 (complete), as previously reported by Septiani et al. [15]. There was no occurrence of FSR in control samples in any of the tested lines, and kernels appeared healthy (Fig. 1A). Conversely, the characteristic pink fungal mycelium of *F. verticillioides* was observed around the germinating seeds of susceptible lines after treatment (Fig. 1A), with different intensity levels depending on the genetic background (Fig. 1B). For convention in this paper, lines with an FSR score below 2.5 are considered resistant. Least Significance Difference (LSD) test (*p* < 0.05) confirmed the presence of two classes of phenotypes: resistant (H99, A632, W153R, HP301) and susceptible (B73, F7, B96, Mo17). FSR scores ranged from 1.8 to 4.3, with a mean value of 2.1 for resistant lines and a mean of 3.7 for susceptible lines (Fig. 1B).

To support FSR scoring, fresh seedling length and weight phenotypes were measured (Fig. S2). Even if the MM founder lines could be divided in two distinct phenotypic groups (resistant and susceptible), the principal component analysis of the phenotypes showed the importance of genetic background (PC1 of 63.4%) over response to the pathogen (PC2 of 29.8%; Fig. S2A). As expected, seedling weight and length were positively correlated (0.6); FSR was negatively correlated with the seedling length (-0.5), but was poorly negatively correlated (-0.1) with the seedling weight (Fig. S2B). Significant negative correlations between these two traits and FSR were previously reported for seedlings of the MAGIC maize population [15], and of a maize diversity panel [17], highlighting the ability of *F. verticillioides* to influence seedling germination and growth at the early stages of inoculation.

### Genetic background is the main source of transcription variation among MM founder lines

**Fig. 2:**
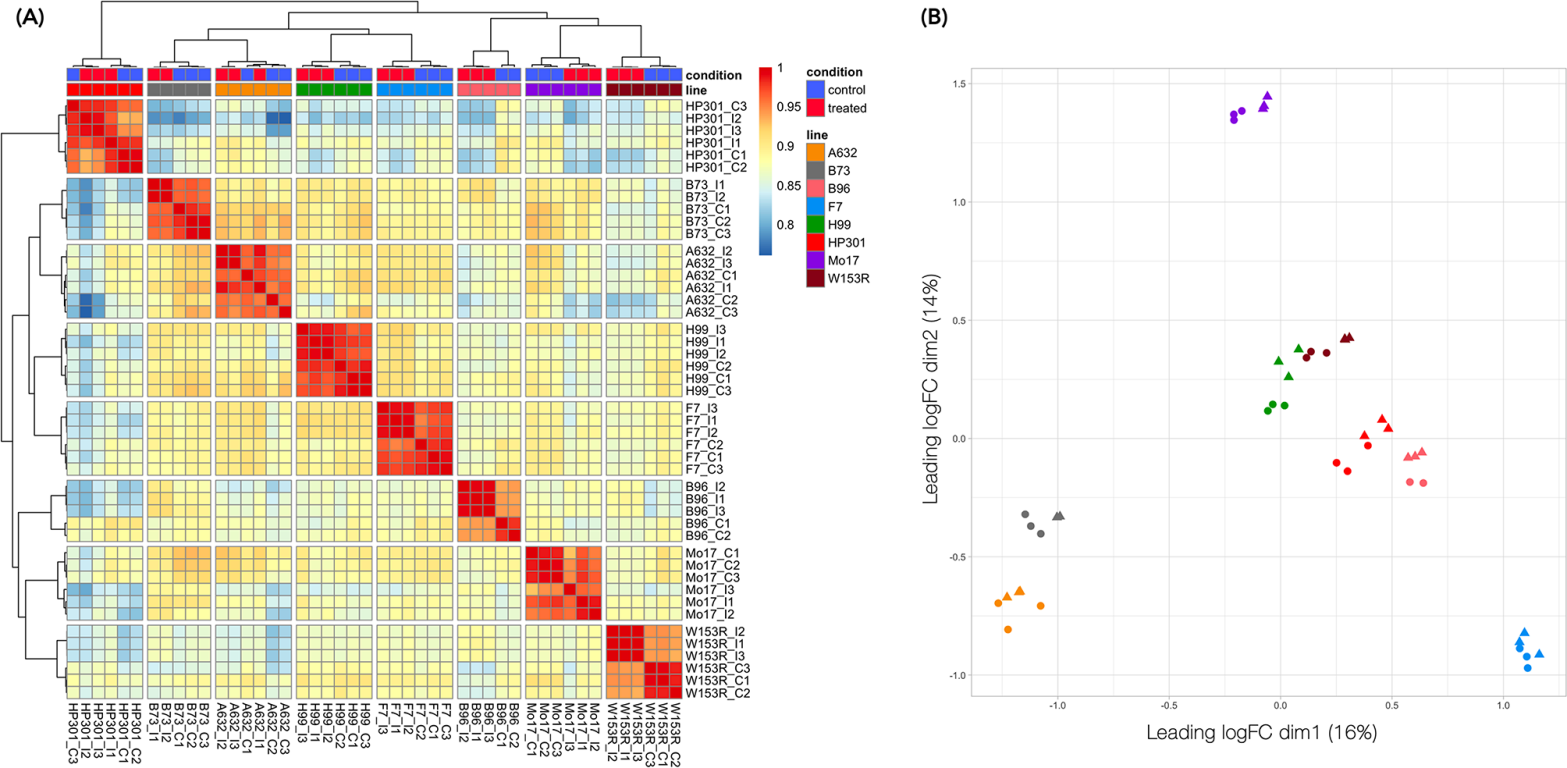
Biological replicate analysis after outlier removal. **(A)** Heatmap correlation of Counts Per Million (CPM) normalized data among biological replicates. **(B)** Multidimensional scaling of normalized expressed genes of each replicate. Colors are given according to the MM founder line. Circles indicate control samples, while triangles indicate treated samples

To obtain a comprehensive understanding of the global expression profile 72 h post- *F. verticillioides* inoculation in maize, the transcriptomes of control and treated samples were paired-end sequenced. We processed a total of 48 samples, including three biological replicates for each condition for each of the eight MM founder lines. In total, more than 2.3 billion reads were generated with an average of 48.6 million reads per sample (ranging from 36.5 to 64.7 million reads), as shown in Table S1. After the quality filtering, raw reads were mapped to the *Zea mays* B73 reference genome v4 [47], with an average mapping rate of 95%, of which 88% mapped uniquely (Table S1.). Gene expression levels of the known annotated genes in the B73 reference transcriptome were quantified within the mapping step. To accurately compare gene expression among samples, we normalized the raw read counts (Table S2) for each individual transcript according to the *edgeR* R package, obtaining the CPM values. Since low-expressed genes constitute noise for the statistics, they were filtered out for the downstream analysis, ending up with a total of 30,800 genes out of 39,389 from the gene annotation on B73 v4. The hierarchical clustering on normalized data identified two sample outliers that were removed (Fig. S3A, B). Without the outliers, the biological replicates within each line showed an acceptable level of correlation (Fig. 2A). Multidimensional scaling of distances of normalized expression levels was in concordance with the principal component analysis of the phenotypes (Fig. S2A): dissimilarity among samples did not show a specific pattern associated to the disease resistance/susceptibility phenotype (Fig. 2B), even if the highly resistant line H99 showed the strongest whole-transcriptome similarity with the second highly resistant line A632, and the weakest similarity with the highly susceptible line Mo17 (Fig. S3C). As expected, the variability within each line was lower than the one between lines. Even if the genetic background of the MM founder lines drives the main differences measured among samples, overall, the positive correlations between MM founder line transcriptomes, suggested the activation of a common pattern caused by the *F. verticillioides* inoculation (Fig. S3C).

### Effects of *F. verticillioides* infection on the transcriptome of each parental line

We applied a generalized linear model (*glm*) to test for the differential expression of the non-normally distributed gene expression data comparing different groups of samples. To answer to the three biological questions previously described, we applied the *glm* statistical test with three different contrasts, obtaining: (i) the genes differentially expressed at 72 hpi within each line; (ii) the genes differentially expressed at 72 hpi and shared between all lines; (iii) the genes significantly up- or down-regulated in the resistant lines (Table S3). For convention in this paragraph, a gene is considered differentially expressed based on a False Discovery Rate (FDR) threshold < 0.05 and on a Log2 Fold Change (FC) > 1 or < -1.

**Fig. 3:**
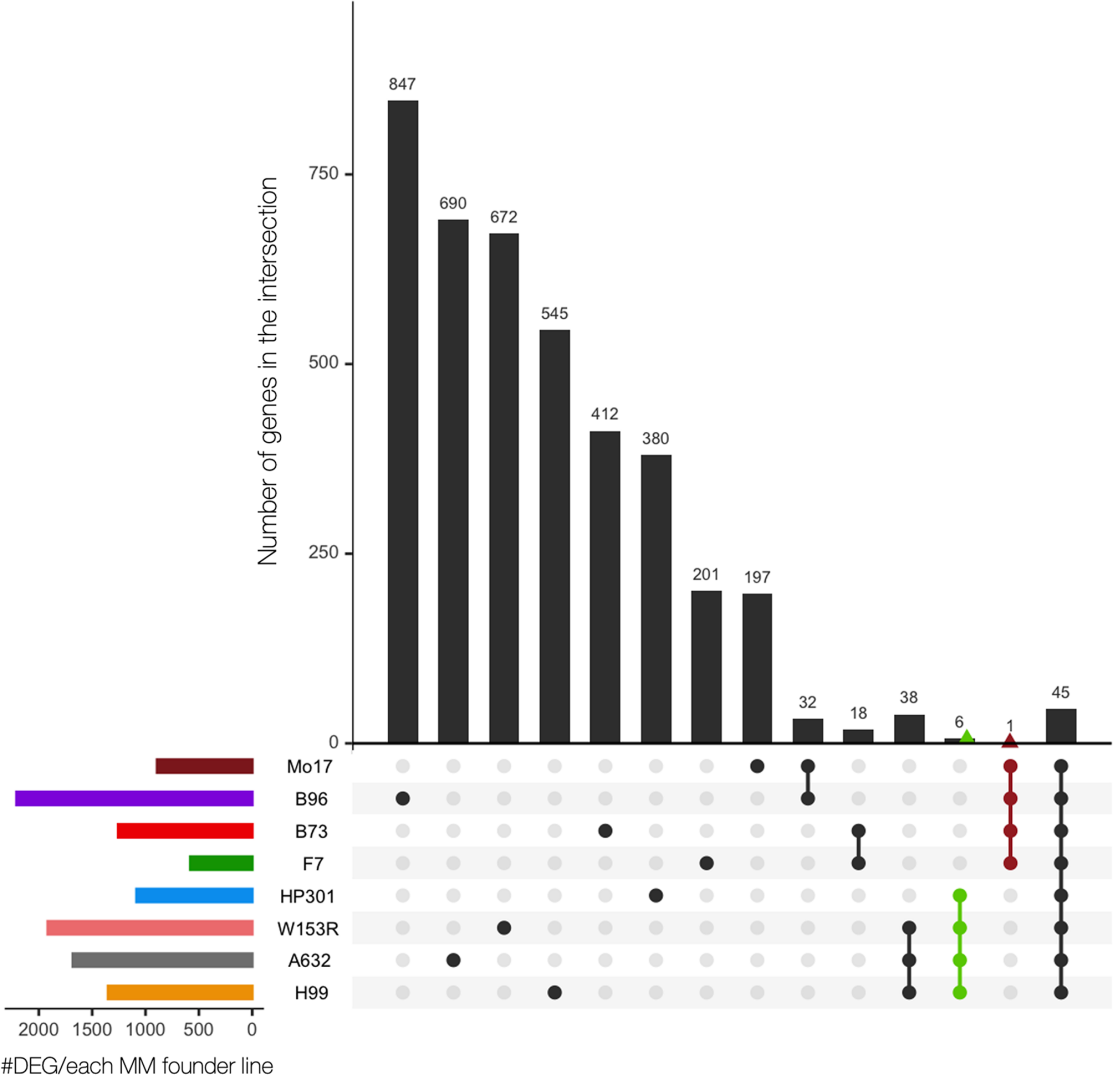
Differentially expressed genes at 72 hpi within each MM founder line. The differentially expressed genes were obtained from contrast 1 comparing control and treated samples within each line. A gene is considered differentially expressed based on a False Discovery Rate (FDR) threshold < 0.05 and on a Log2 Fold Change (FC) > 1 *or < -1*. Green and red colors refer to the groups of the four resistant and the four susceptible founder lines, respectively

*Biological question 1* – Transcriptional perturbations induced by *F. verticillioides* inoculation in different maize lines. A total of 10,965 genes were significantly expressed in at least one line when comparing the two conditions within each line. Only 45 genes were in common among all the MM founder lines, and, interestingly, all up-regulated (Fig. 3). The small number of shared genes is not surprising, considering the footprint of the different genetic backgrounds on the gene expression profile of the lines. The largest proportion of these common genes was related to the disease resistance processes, which included nine DEGs involved in the secondary metabolism, namely terpene biosynthesis, as three terpene synthases (*Zm00001d041082*, *Zm00001d024208* and *Zm00001d029648*) and two cyclases (*Zm00001d032858* and *Zm00001d024210*), ten resistance genes encoding proteinase inhibitors, thaumatin and pathogenesis-related proteins, seven cytochrome P450 oxidoreductases, three and two DEGs associated to lipid and cell wall metabolism, respectively, such as fatty acid desaturases, xyloglucan fucosyltransferase and glycoside hydrolase, and one WRKY transcription factor (*Zm00001d009939*) (Table S3). The discussion about most of these DEGs is postponed to the paragraphs below.

The MM founder line B96 displayed the highest number of differentially expressed genes (2,209), while F7 the lowest (579).

*Biological question 2* – Transcriptional perturbations induced by *F. verticillioides* inoculation in *Zea mays*. The second biological question considers only the inoculation effect, regardless the genetic makeup of the lines. In this analysis, a total of 921 genes were significantly up- or down-regulated in the treated samples.

*Biological question 3* – Transcriptional perturbations induced by *F. verticillioides* inoculation in resistant lines. When grouping resistant and susceptible lines and testing for DEGs between the two groups, we found 3,514 genes significantly up- or down-regulated.

### Gene Ontology enrichment shows that terpene pathway is highly enriched

We performed a gene ontology (GO) enrichment analysis comparing control and treated samples for each of the tested MM lines. Regardless of the basal transcriptomic differences between lines, the analysis across all genotypes reported enrichment in categories with suggestive function in relation to fungal infection. Metabolic processes associated with terpene synthesis were found to be highly enriched in all genotypes (Fig. S4). A huge array of terpenoid phytoalexins, including diterpenoids (kauralexins and dolabralexins) and sesquiterpenoids (zealexins), are abundantly accumulated in maize in response to multiple fungal pathogens to inhibit their growth [48, 49]. Previous transcriptomic studies reported that terpene synthase genes were highly expressed in response to *F. verticillioides* [18, 31]. Moreover, an increased susceptibility to *F. graminearum* was detected in maize lines defective in kauralexins or zealexins [50], whereas mutants silenced in terpene synthases were more susceptible to *Ustilago maydis* [51]. Further enriched categories were Oxidoreductase activity (all MM lines), Defense response (in lines B96, HP301, and W153R), Defense response to fungus (F7 and Mo17), Chitin metabolic/catabolic process (B73, Mo17 and W153R), Lignin metabolic/catabolic process (B96, Mo17 and W153R), and Lipid metabolic/biosynthetic process (HP301, Mo17 and W153R; Fig. S4). Chitin is a well-known elicitor of immune responses in plants, and it is generally assumed that chitin oligomers are released during pathogen ingress and are recognized by plants [52]. Among the plants’ reactions to chitin are included: protein phosphorylation, chitinase and glucanase activation, generation of reactive oxygen species, biosynthesis of jasmonic acid and phytoalexins, lignification and cell wall thickness [53, 54]. Additionally, lipids play a key role at various stages of host–pathogen interactions in determining virulence and modulating plant defense [55].

Our findings indicated that all these processes were essential during *F. verticillioides* infection in the MM lines, as already observed in response to the same fungus in seedling [18] and ear [31] as well as in other pathosystems [44, 56, 57]. It is notable that Response to auxin was a specific GO pathway enriched in the highly resistant line H99. Several genes related to auxin were strongly enriched in maize in response to *U. maydis* [58]. Moreover, in the soybean–*Phytophthora sojae* interaction, auxin accumulated to a greater extent in a relatively resistant soybean cultivar [59].

### Weighted gene co-expression network analysis identifies cluster of genes highly correlated with FSR severity

Once the differential expression gene analysis was used to identify lists of genes answering each of the three biological questions, a correlation approach was implemented to identify clusters of highly correlated genes (modules) associated with the FSR severity. Since the main factor of transcriptional (dis)similarity was driven by the intrinsic genetic makeup of the MM founder lines and not by the resistance condition (Fig. 2), we needed to remove the background noise. For this reason, we used only the differentially expressed genes selected based on statistical significance, regardless of the magnitude of the difference. Therefore, we used the 20,166 differentially expressed genes coming from the sum of all the three contrasts described in the previous paragraph. Moreover, since the empirical Bayes procedure implemented in *edgeR* normalization shrinks the dispersion of the expression data towards a consensus value, we filtered out genes that had no variance, ending up with 20,042 genes. To identify important genes up- or down-regulated after *F. verticillioides* inoculation, we used a treatment-specific approach, building a network in both the control and treatment conditions separately. Each network contains 23 samples (3 replicates per 8 MM founder lines, without one outlier for each treatment) and 20,042 genes (putative network nodes). The network built with the control samples hereafter will be called *Control-Network*, while the network built only with treated samples will be called *Treated-Network.* All possible pairwise correlations were calculated in parallel for the 20,042 genes in both conditions and converted into measures of connection strength by ranking their absolute value to a power (see *Methods* paragraph).

**Fig. 4:**
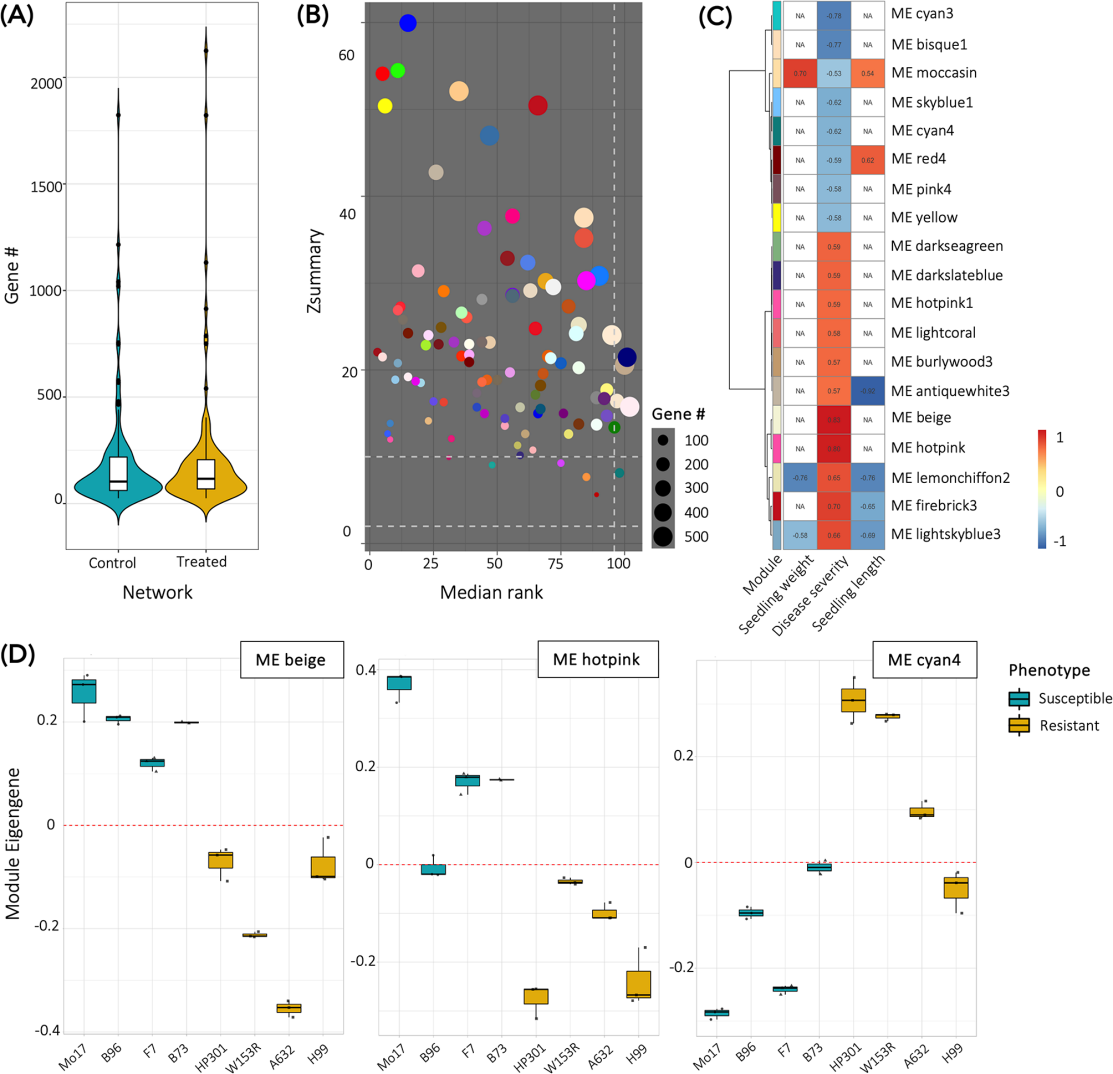
Characteristics of the network construction. **(A)** Distribution of the module dimension. **(B)** The median rank and the *Zsummary* statistics in the module preservation analysis between *Control-Network* (reference) and *Treated-Network* (test). **(C)** Correlation Module Eigengene (ME) with traits in the *Treated-Network*. **(D)** Differentially expressed modules from *Treated-Network* between the resistant and the susceptible phenotypes

Genes with similar patterns of connection strength were clustered together in a module of co-expressed genes. On this basis, we found 102 modules for the *Control-Network*, and 97 modules for the *Treated-Network* (Fig. 4, Table S4). The sum of the connection strength of a gene is called connectivity, and it is a key property that defines how frequently a gene interacts with the other genes. In a biological network, the sum of the connection strength of each gene (node) across the whole network represents how strongly the gene relates to all the others. We based the network analysis on the hypothesis that the pathogen treatment would have disrupted the gene-gene interaction observed in the *Control-Network*, thus changing the network properties. This step was important because it highlights whether the relationships (connectivity patterns) and correlation structures between expressed genes composing each module are preserved or interrupted when measured in a different dataset [60]. For this study, we set the *Control-Network* as the reference, while we considered the *Treated-Network* as the test. In general, 95 *control* modules out of 102 showed high level of preservation (*Zsummary* > 10), whereas seven modules showed moderate to low evidence of preservation (2 < *Zsummary* < 10) (Fig. 4B). The modules exhibit high *medianRank* reinforcing the fact that they were poorly preserved. The disrupted modules in the control reference network (*Control-Network*) contained 667 genes, that had a significant enrichment in anion binding and small molecular binding. The modules contained 19 hub genes, among which the top hub gene was one heat shock transcription factor (*Zm00001d016255*).

The following analysis was based on the gene co-expression modularity property, with the assumption that genes highly interconnected within the network are usually involved in the same biological pathway. Under this assumption, modules coming from the *Treated-Network*, were correlated with the FSR severity score evaluated in the RTA samples at 72 hpi. The Pearson correlation showed that 19 out of 97 modules were significantly associated with the severity phenotype (*p* < 0.01) (Fig. 4C). Since there were modules related to FSR, we hypothesized that among them, modules related to the resistant phenotype were also included. To test our hypothesis, we run a linear model on the Module Eigengene of each module, which can be considered representative of the expression profile of genes within the module. The statistical test showed that three modules with high correlation (*cyan4*,* beige*,* hotpink*) were also differentially expressed between resistant and susceptible phenotypes (Fig. 4D). The three modules all together contained 363 genes.

To formally test whether differentially expressed genes are less likely to be hub nodes in the co-expression networks, we examine the connectivity distributions. According to the literature, genes with absolute module membership value over 0.9 (to the respective module) are defined as hub genes [21]. The Module Membership is defined as the correlation between an individual gene and the module eigengene. Since we have a lot of modules with a small number of genes, we will expect a high amount of hub genes. We found 200 hubs in the *Control-Network* and 201 hubs in the *Treated–Network*. To find genes with potential relevance to resistance against *F. verticillioides*, we decided to focus on the hub genes in the *Treated–Network* belonging to the 19 modules significantly associated with the severity phenotype with a correlation higher than 0.6. We obtained 91 hub genes and interestingly, some of them showed a close relationship to disease resistance (Table S5). For example, *Zm00001d029711* and *Zm00001d030995* encode for the basic leucine zipper (bZIP)-transcription factors 78 and 111, respectively. *Zm00001d029711* was significantly down-regulated in the A639, H99, HP301, and B96 MM founder lines (biological question 1) and in response to *F. verticillioides* on the whole (biological question 2), whereas *Zm00001d030995* was significantly upregulated only in the F7 line. *Zm00001d022442* encodes for a further bZIP-transcription factor (58), but this gene was not found to be differentially expressed. Several studies have described the role of transcription factors that contain a bZIP domain [61], and various processes, such as abiotic stress response, seed maturation, flower development and pathogen defense, are regulated by bZIP family members [62]. The modulation of distinct *ZmbZIP* genes was previously reported following four different fungal infections with *U. maydis*, *Colletotrichum graminicola*, *F. moniliforme* and *Sphacelotheca reiliana* [63]. Moreover, transgenic soybean plants overexpressing bZIP15 showed increased resistance against *Sclerotinia sclerotiorum* and *P. sojae* [64].

*Zm00001d032240* encodes for the MYB-transcription factor 146 and was observed significantly upregulated in the A632, B73, B96, and Mo17 founder lines and in response to *F. verticillioides* on the whole (biological question 2). Interestingly, the same gene was specifically induced in the CO354 susceptible genotype in response to FER [31].

Two glycoside hydrolases (*Zm00001d029154* and *Zm00001d029164*) were differentially modulated in A639 and B73 MM lines, and found among the genes specifically responsible for the maize resistance to *F. verticillioides* (biological question 3). These enzymes are involved in the metabolism of various carbohydrates, which in plant pathogenic fungi was directly associated with the degradation of plant cell wall [65]. It was previously reported that when *F. verticillioides* attacks maize plants, carbohydrate metabolism is one of the main metabolic processes to be affected [18, 66]. This suggests that the pathogen could force the plant to produce specific carbohydrates as food sources.

*Zm00001d048327* and *Zm00001d048329* both encode adenosine triphosphate (ATP)-binding cassette (ABC) transporters and were induced in W153R and Mo17. Additionally, *Zm00001d048327* was also upregulated in H99, B73, B96 and in response to *F. verticillioides*. ABC transporters guide the exchange of chemically diverse substances across cellular membranes using ATP as an energy source [67], and mediate the detoxification processes of both internal and external xenobiotics, confining them internally in vacuoles or in apoplastic regions [68]. An ABC transporter was previously identified as differentially expressed in response to Gibberella ear rot in maize kernels of resistant and susceptible inbreds [69]. Moreover, the same transporter was demonstrated to confer resistance to multiple fungal pathogens in wheat [70].

*Zm00001d007181* encodes for a calcium-binding protein and was differentially modulated in W153R and B96 MM founders, and in resistant lines (biological question 3). He and co-workers [71] detected a hub gene encoding for a similar protein responding to maize gray leaf spot caused by *Cercospora zeina*. Furthermore, mutations in the calcium-binding protein in wheat determined resistance to Fusarium head blight [72].

The hub gene for a fatty acid hydroxylase (*Zm00001d011765*) was significantly upregulated in resistant lines (biological question 3). The hydroxy-fatty acid production is mainly the result of enzymatic reactions catalyzed by cytochrome P450-dependent fatty acid hydroxylases [73]. These compounds are among the most studied oxylipins for their strong antifungal activity against a large number of fungi [74]. Additionally, they were connected to further biological functions, such as signaling, virulence, and response mechanisms towards stress factors [74].

In summary, the predicted functions of hub genes established that transcription factors, carbohydrate metabolism, solute transport processes, calcium signaling, and lipid pathways played a central function in FSR resistance.

### Integration of QTL mapping and differentially expressed genes reveals further candidates for FSR resistance

In previous QTL mapping, three FSR-resistance QTL (qFSR4.1, qFSR4.2 and qFSR5.1) were identified in chromosomes 4 and 5 [15]. Using the physical location of QTL, we screened for the presence of differentially expressed genes within the three QTL regions and we found 7 and 14 genes in the QTL qFSR4.1 and qFSR5.1, respectively (Table S6). No differentially expressed genes were detected in the QTL qFSR4.2. Among genes associated with these intervals of interest, 7 were analyzed in more detail based on their known defensive role: a glycoside hydroxylase (*Zm00001d048669*), a pectin lyase (*Zm00001d048696*), a laccase (*Zm00001d048658*), a APETALA 2/ethylene responsive element binding protein (AP2/ERF) transcription factor (*Zm00001d016616*), two negative regulators of plant-type hypersensitive response (*Zm00001d016584* and *Zm00001d016585*) and a heat shock protein (HSP; *Zm00001d016674*).

In the region containing the qFSR4.1 QTL, we observed the upregulation of two genes (*Zm00001d048669* and *Zm00001d048696*) related to carbohydrate metabolism and degradation of cell walls, highlighting once again the central role of this pathway. In the same genomic region, the gene *laccase* was differentially modulated in the B73 founder line and in resistant lines (contrast 3). In plants, laccase catalyzes monolignols oxidation and participates in lignin polymer formation [75]. It has been broadly reported that phenylpropanoid pathway is involved in the plant defense response against abiotic and biotic stresses, mainly through activating the biosynthesis of secondary metabolic compounds, such as flavonoids, lignin, hydroxycinnamic acid, and terpenoids [76]. These pathways were strongly represented and induced in a maize resistant genotype in response to FER [31].

In the genomic region containing the qFSR5.1 QTL, the AP2/ERF transcription factor was induced in the B96 and W153R MM lines. The same two lines showed a downregulation of HSP (*Zm00001d016674*) along with H99 and HP301. Moreover, HSP was down-regulated in the contrast analyzed in biological question 3. This agrees with previous results reported by Maschietto and co-workers [8] that also found several HSPs and AP2/ERF transcription factors in two overlapping QTL for resistance to FER and fumonisin contamination.

Two additional interesting genes encoding for negative regulators of plant-type hypersensitive response (*Zm00001d016584* and *Zm00001d016585*) were found in qFSR5.1 QTL and significantly induced in B73 and H99, respectively. Plant hypersensitive defense response is a rapid localized cell death that limits pathogen spread and is associated with other responses, including ion fluxes, an oxidative burst, lipid peroxidation and cell wall fortification [77]. These two genes would therefore be interesting candidates for further study in relation to the response to infection by *F. verticillioides* and other pathogens.

## Conclusions

The current study characterized the plant response to Fusarium seedling rot disease in maize using gene expression profiling of eight divergent maize MAGIC founder lines with contrasting levels of resistance. RNA sequencing approach identified several hundred differentially expressed genes, whose functions were explored through Gene Ontology analysis that highlighted a strong enrichment in metabolic processes associated with terpene synthesis. A WGCNA further refined the pool of genes with potential implications in disease response and found a limited set of hub genes such as those encoding bZIP and MYB transcription factors, or involved in carbohydrate metabolism, solute transport processes, calcium signaling and lipid pathways. Finally, combining transcriptomic data with previous QTL mapping, additional gene resources were provided that could be studied to evaluate their usefulness in marker assisted selection. The characterization of mutants generated in our laboratory editing and overexpressing candidate genes derived from this, and our previous research is already ongoing to claim the important role of favorable genetic variants in the breeding of maize for *F. verticillioides* resistance.

## Electronic supplementary material

Below is the link to the electronic supplementary material.

Supplementary Material 1: **Figure S1.** Evaluation of* F. verticillioides* inoculation steps in a time-course experiments (48-168 hpi) in the highly susceptible Mo17 and in the highly resistant H99 MM founder lines. **Figure S2. **Phenotype analysis of traits measured by the Rolled Towel Assay. **Figure S3.** Transcriptomic data exploration for outlier removal.

Supplementary Material 2: **Figures S4**. Gene Ontology enrichment analysis of differentially expressed genes between treated and control samples within each MM line.

Supplementary Material 3: **Methods S1**. Contrasts description applied through *edgeR* for each biological question.

Supplementary Material 4: **Table S1**. Statistics of paired-end sequenced reads produced, along with the total number of aligned reads on the B73 Reference Genome v4, for each biological replicate. C, control; I, inoculated.

Supplementary Material 5: **Table S2**. Raw counts for each gene in each biological replicate (indicated by number 1, 2, 3) across the eight MAGIC maize founder lines. C, control; I, inoculated.

Supplementary Material 6: **Table S3**. Differentially Expressed Genes analysis obtained using the *edgeR* R package. Each sheet addresses the three biological questions raised in this study. In *DEG* columns, it is indicated whether a gene is differentially expressed based on the convention of this analysis, where a gene is considered differentially expressed if it meets statistical significance (FDR < 0.05) and a Fold Change threshold (|log_2_FC| > 1).

Supplementary Material 7: **Table S4**. Network properties for *Control-Network* (first sheet) and *Treated-Network* (second sheet). MM, Module Membership, *i.e*. the correlation of the gene expression profile with the module eigengene of the given module; kTotal, whole network connectivity, defined as the sum of connection strengths of a gene with the other network genes, in other words it measures how correlated a gene is with all other network genes; kWithin, intramodular connectivity, which measures how connected, or co-expressed, a given gene is with respect to the genes of a given module; kOut = kTotal - kWithin; kDiff = kWithin - kOut; GS.severity = Gene Significance for the severity phenotype, *i.e.* correlation between the gene expression and the trait.

Supplementary Material 8: **Table S5**. List of the 91 hub genes in the *Treated–Network,* belonging to the 19 modules significantly associated with the severity phenotype and showing a correlation greater than 0.6. For the meaning of each column, refer to the caption of Table S4.

Supplementary Material 9: **Table S6**. List of putative candidate genes for *Fusarium verticillioides* resistance in the genomic regions containing QTL for FSR.

## Data Availability

The raw reads generated and analyzed during the current study are available on the Sequence Read Archive database of National Center for Biotechnology Information (https://www.ncbi.nlm.nih.gov/sra/) repository under the BioProject accession number PRJNA1063708. Scripts are available from the corresponding author on reasonable request.

## Abbreviations

CPM: Counts per million
DEG: Differentially expressed gene
FC: Fold change
FDR: False discovery rate
FER: Fusarium ear rot
FSR: Fusarium seedling rot
FStR: Fusarium stalk rot
GLM: Generalized linear model
GO: Gene Ontology
GWAS: Genome-wide association studies
hpi: Hours post inoculation
LSD: Least Significance Difference
MAGIC: Multi-parent advanced generation inter crosses
MM: MAGIC maize
PRm: Pathogenesis-related maize
QL: Quasi-likelihood
QTL: Quantitative trait loci
RNA-seq: RNA sequencing
RTA: Rolled towel assay
RT-qPCR: Real-time reverse transcription-quantitative PCR
WGCNA: Weighted gene co-expression network analysis
