# Supplementary material for: Transcriptome profiling of eight Zea mays lines identifies genes responsible for the resistance to Fusarium verticillioides
Source: BMC Plant Biol. 2024 Nov 21;24:1107. doi: 10.1186/s12870-024-05697-y (PMC11580207; doi:10.1186/s12870-024-05697-y)
Supplement: Supplementary file 1 — Supplementary Material 1: Figure S1. Evaluation of F. verticillioides inoculation steps in a time-course experiments (48-168 hpi) in the highly susceptible Mo17 and in the highly resistant H99 MM founder lines. Figure S2. Phenotype analysis of traits measured by the Rolled Towel Assay. Figure S3. Transcriptomic data exploration for outlier removal. [file 12870_2024_5697_MOESM1_ESM.pdf]

Supplementary Figures:

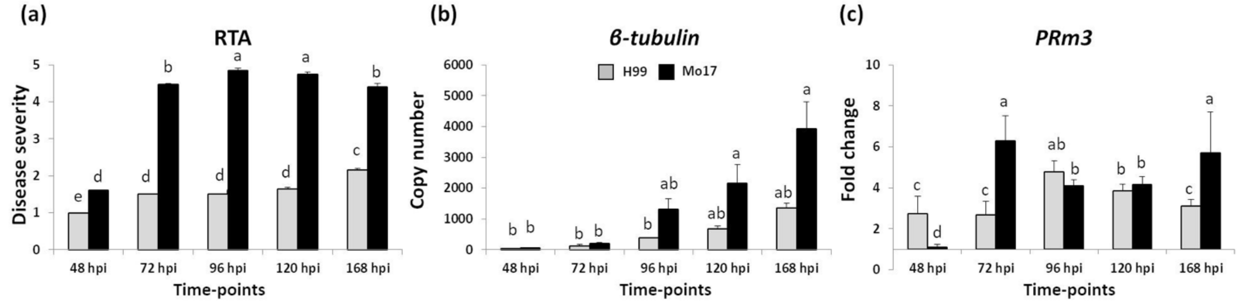

Fig. S1. Evaluation of *F. verticillioides* inoculation steps in a time-course experiments (48-168 hpi) in the highly susceptible Mo17 and in the highly resistant H99 MM founder lines. **(a)** FSR disease severity score was evaluated in seedlings by the Rolled Towel Assay (RTA). A score was assigned according to the level of infection ranging from 1 to 5 (Septiani *et al.*, 2019), where score 1 indicates minimum or no effects (resistant phenotype), while score 5 means that the seed is severely affected (susceptible phenotype). **(b)** Copy number of transcripts for the constitutive *F. verticillioides β-tubulin* gene quantified by RT-qPCR. **(c)** Relative quantification of the expression of maize *PRm3* gene measured by RT-qPCR. On panels (a), (b) and (c), standard deviations (SD) of the mean are indicated by vertical bars (n = 3). Letters on the bars are significantly different at $p<0.05$ in the LSD test.

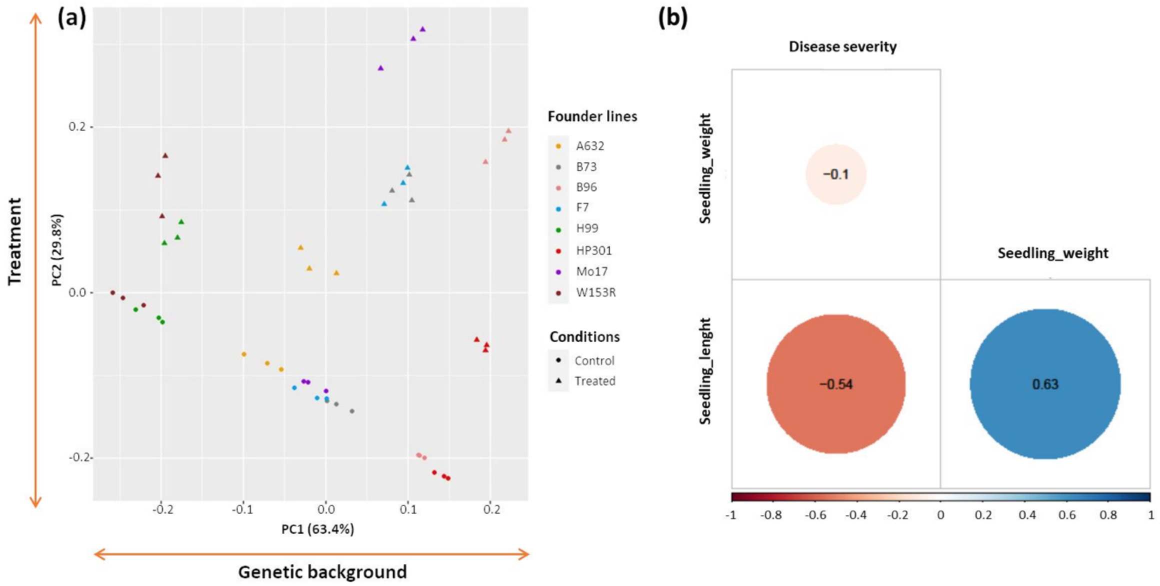

Fig. S2. Phenotype analysis of traits measured by the Rolled Towel Assay. **(a)** Principal Component Analysis of the traits for each biological replicate (seedling length, seedling weight, and FSR disease severity). **(b)** Correlation analysis between phenotypes among all samples.

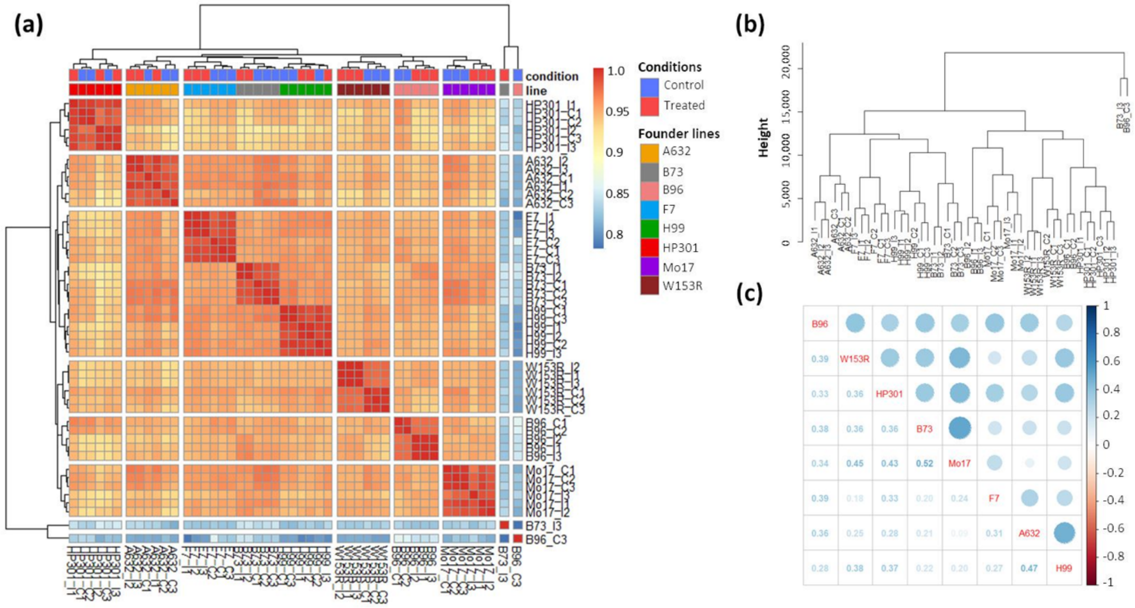

Fig. S3. Transcriptomic data exploration for outlier removal. **(a)** Correlation of Count Per Million (CPM) measurements among biological replicates without removing outliers. **(b)** Unsorted dendrogram of CPM data calculated for each biological replicate. **(c)** Correlation of normalized expression data among the eight MM founder lines.
