# Supplementary figures and images for: Transcriptome profiling of eight Zea mays lines identifies genes responsible for the resistance to Fusarium verticillioides
Source: BMC Plant Biol. 2024 Nov 21;24:1107. doi: 10.1186/s12870-024-05697-y (PMC11580207; doi:10.1186/s12870-024-05697-y)

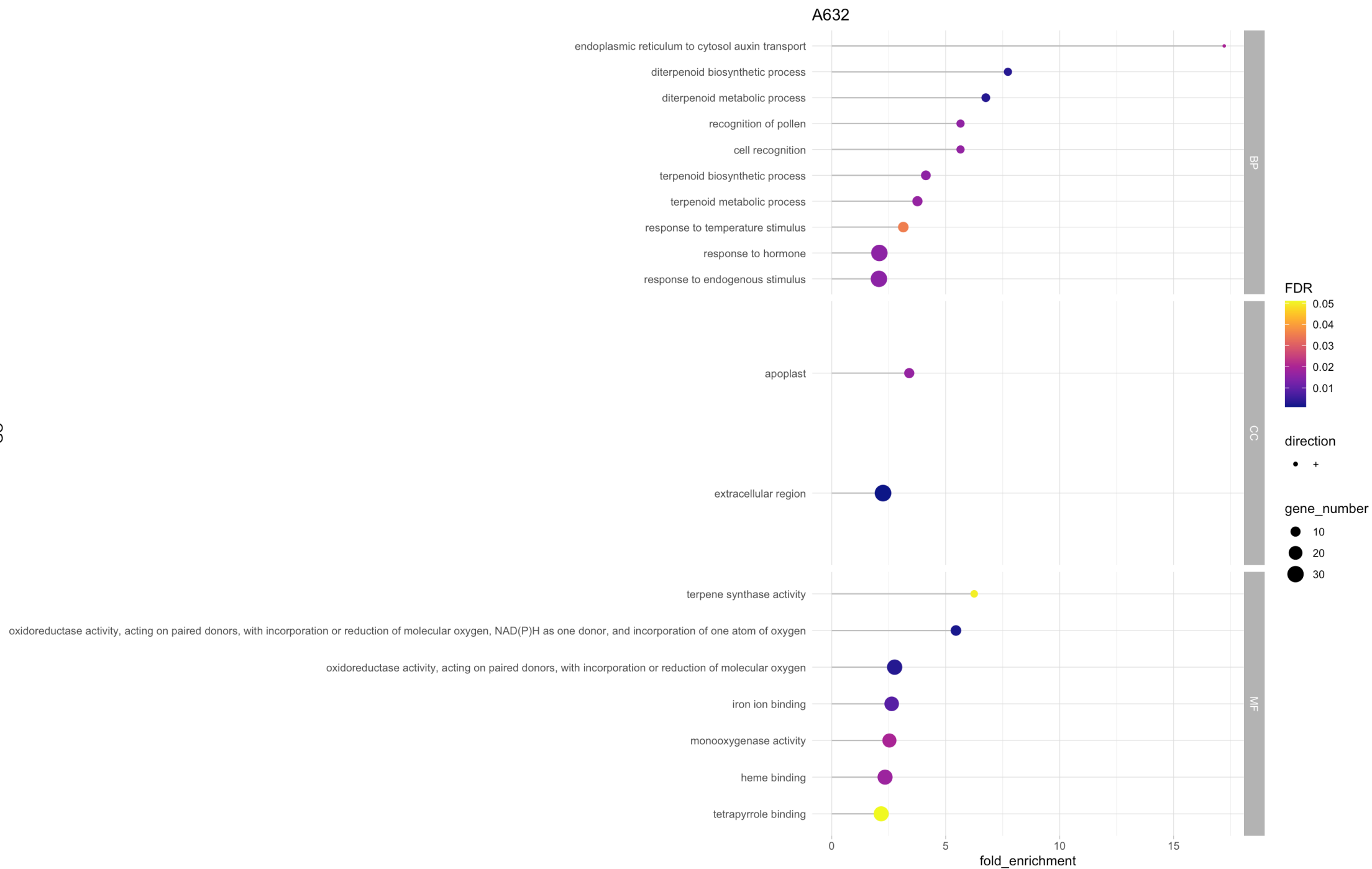

B73

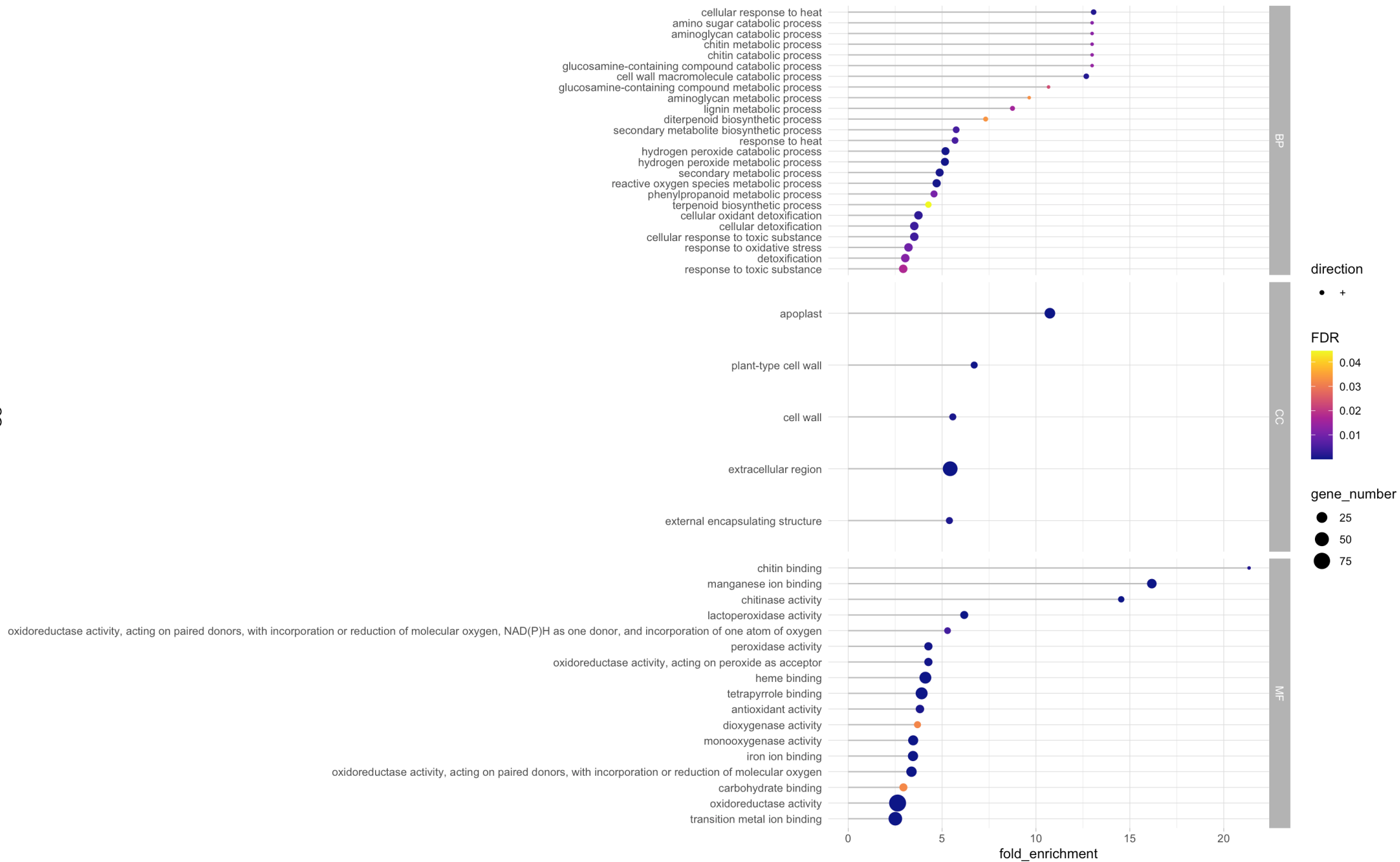

B96

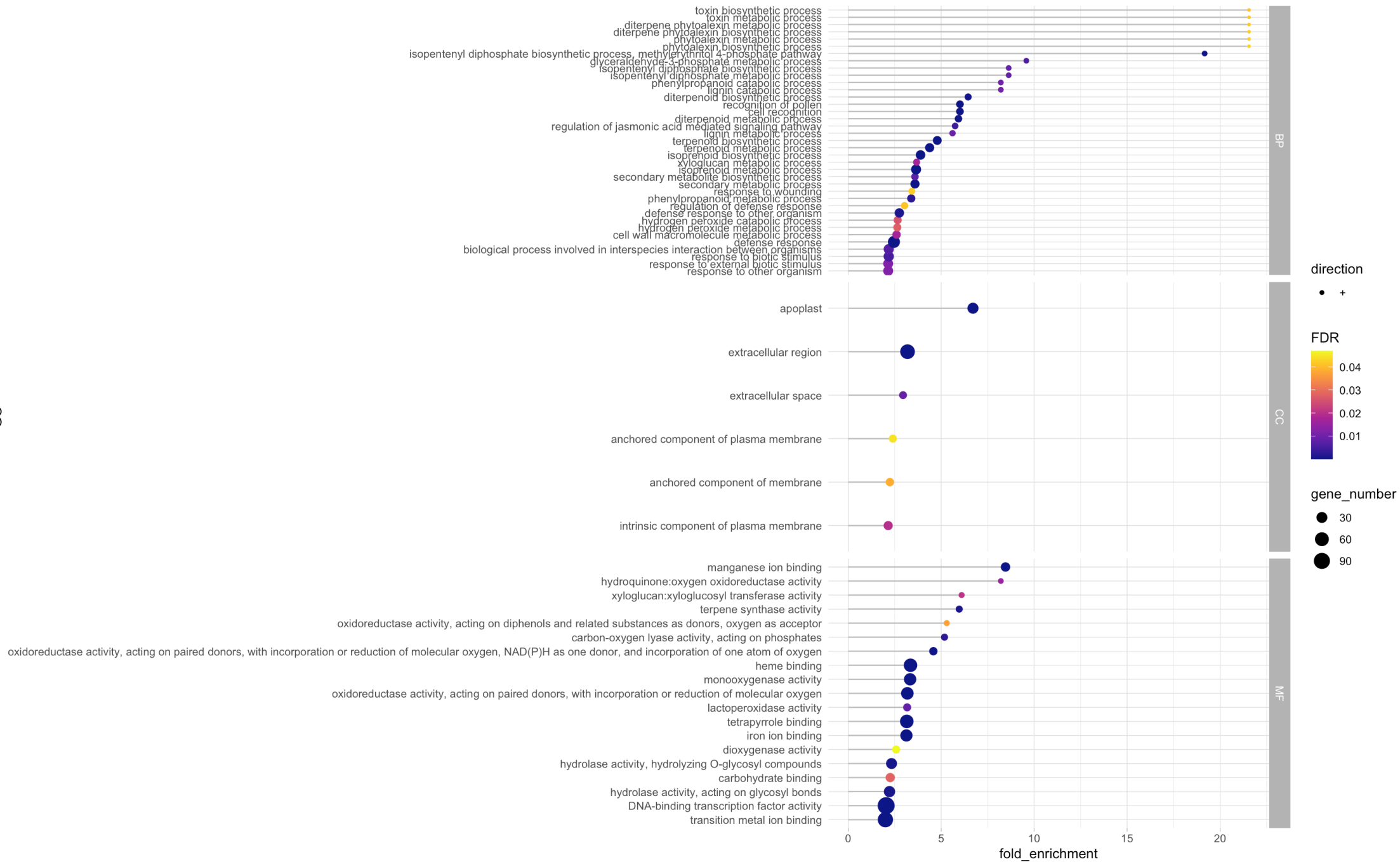

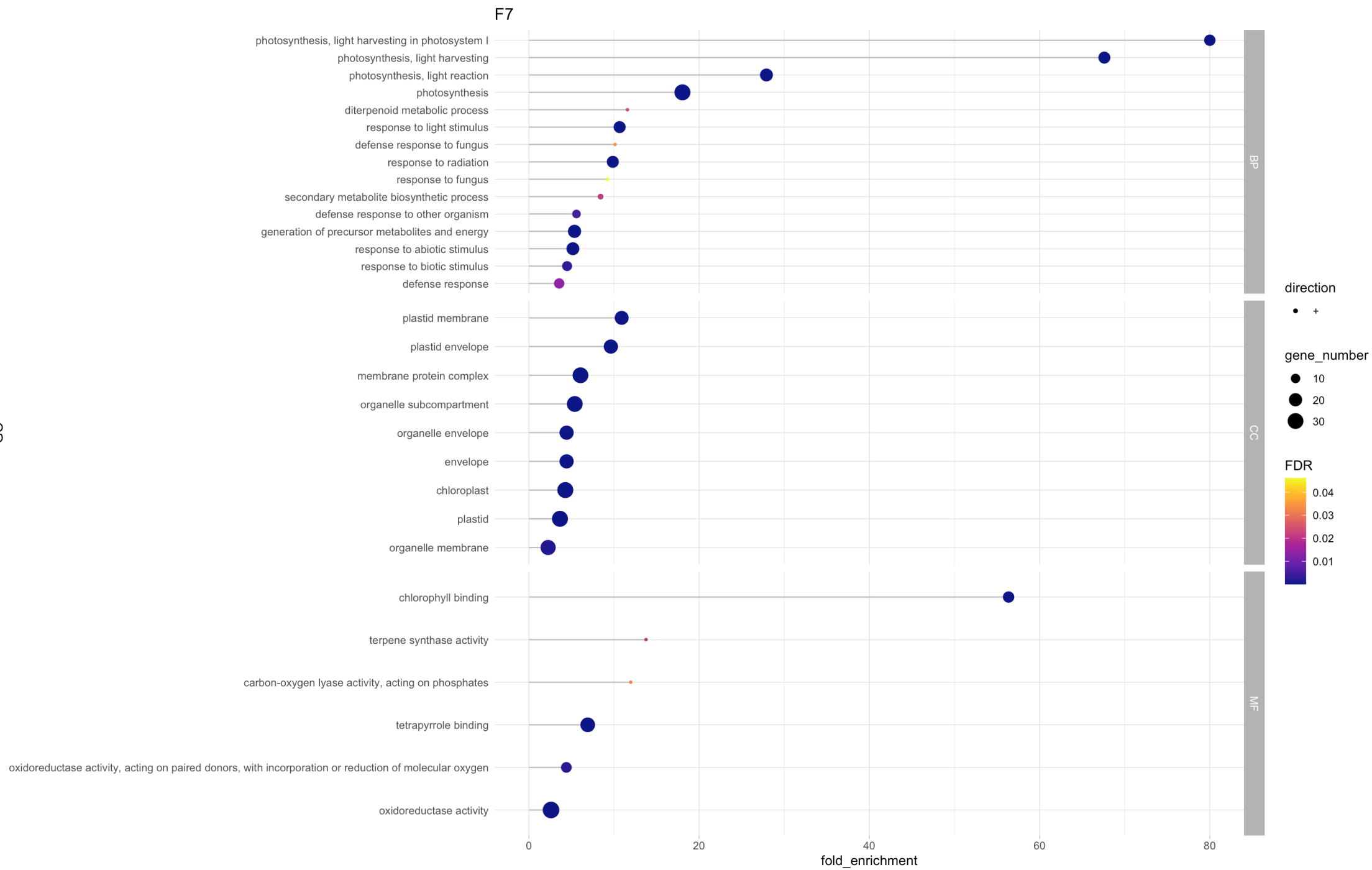

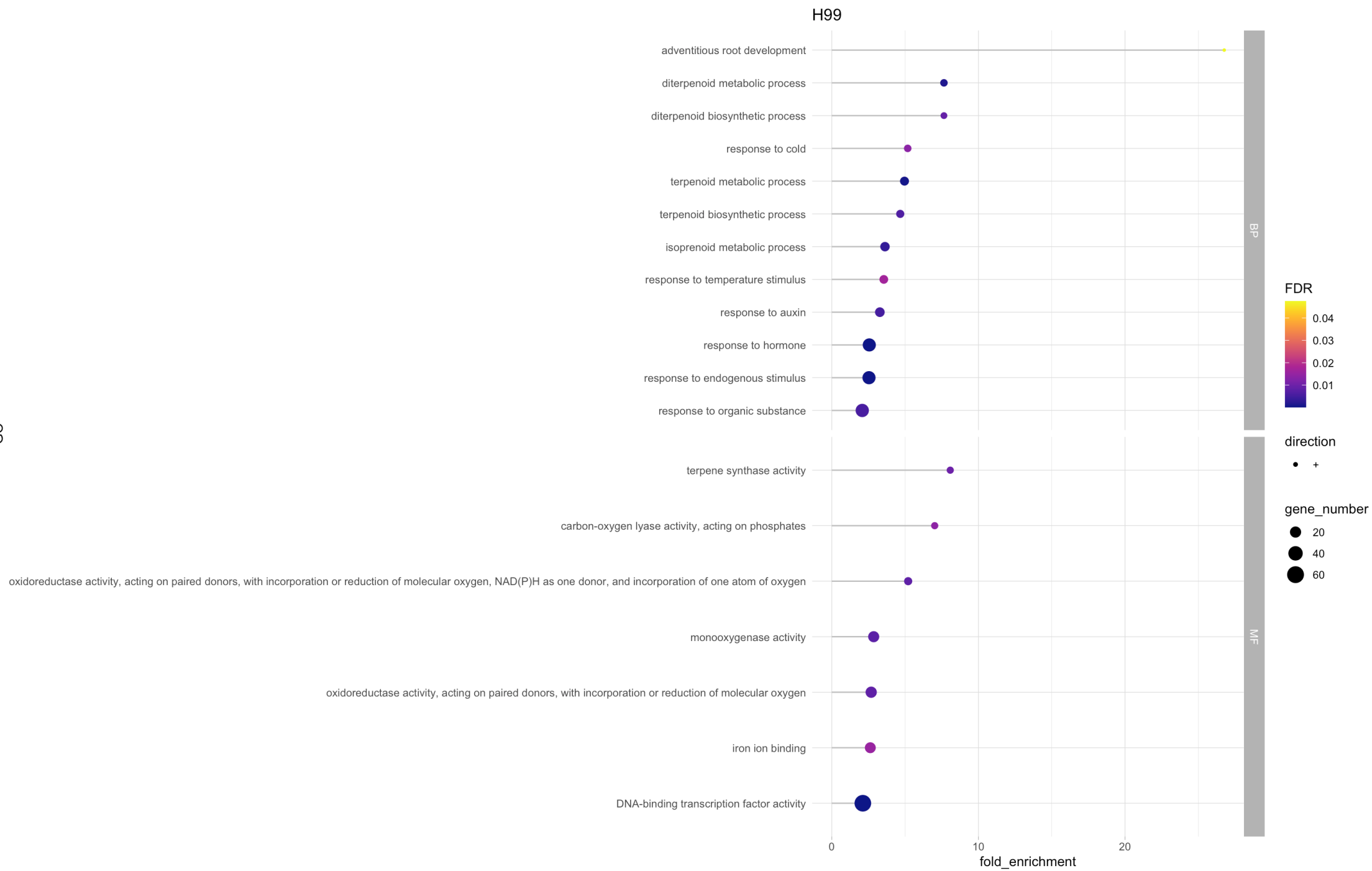

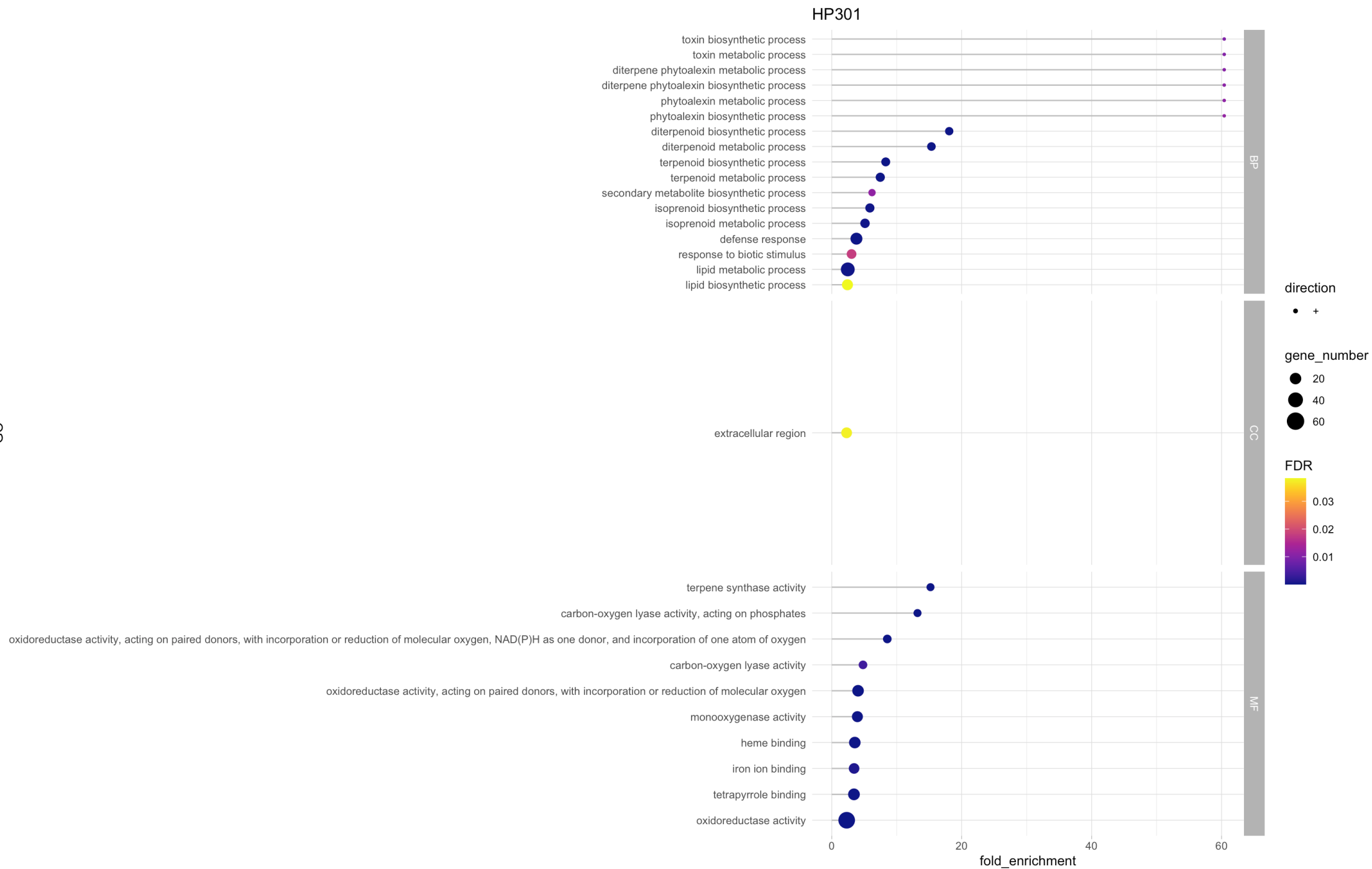

Mo17

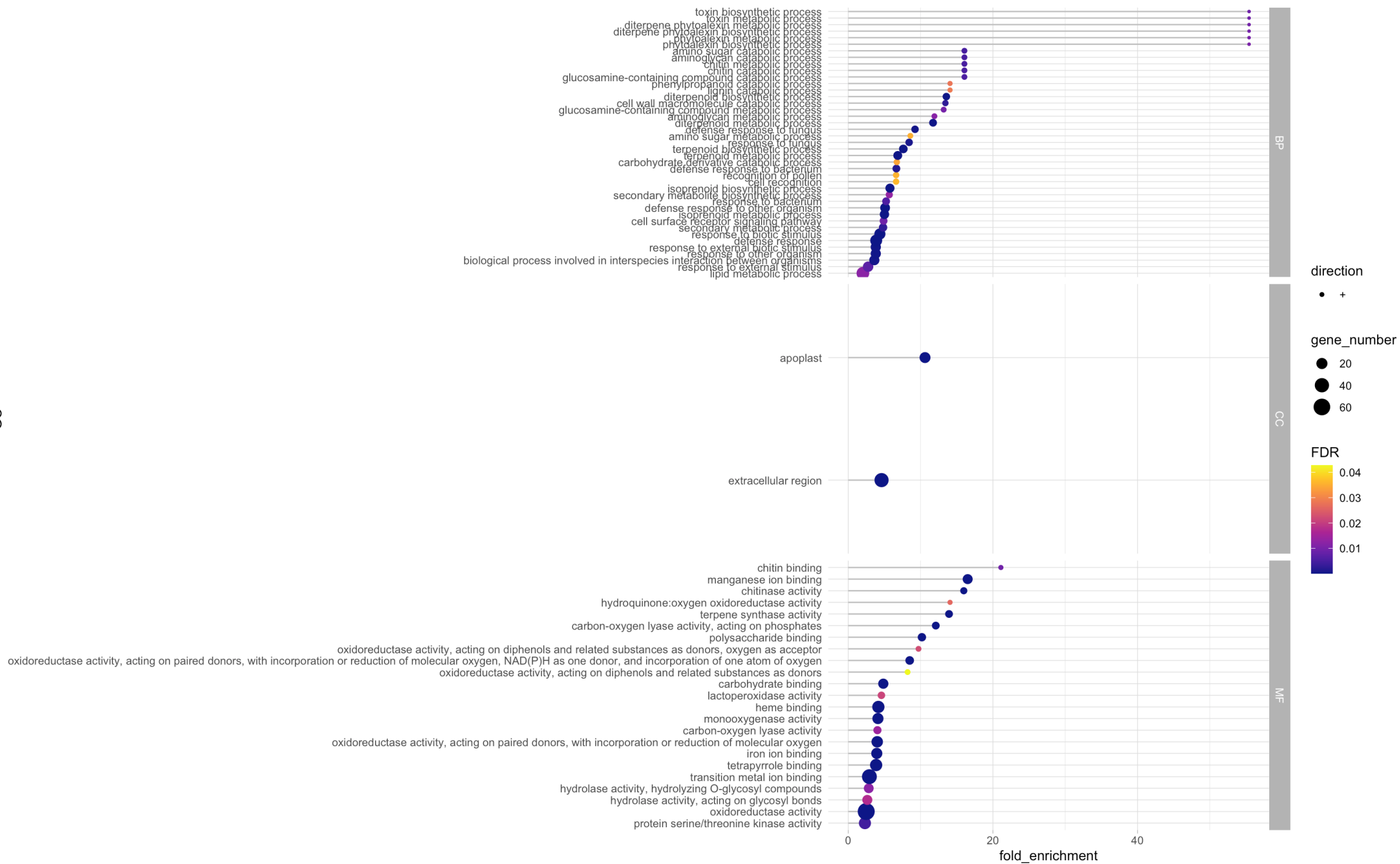

W153R

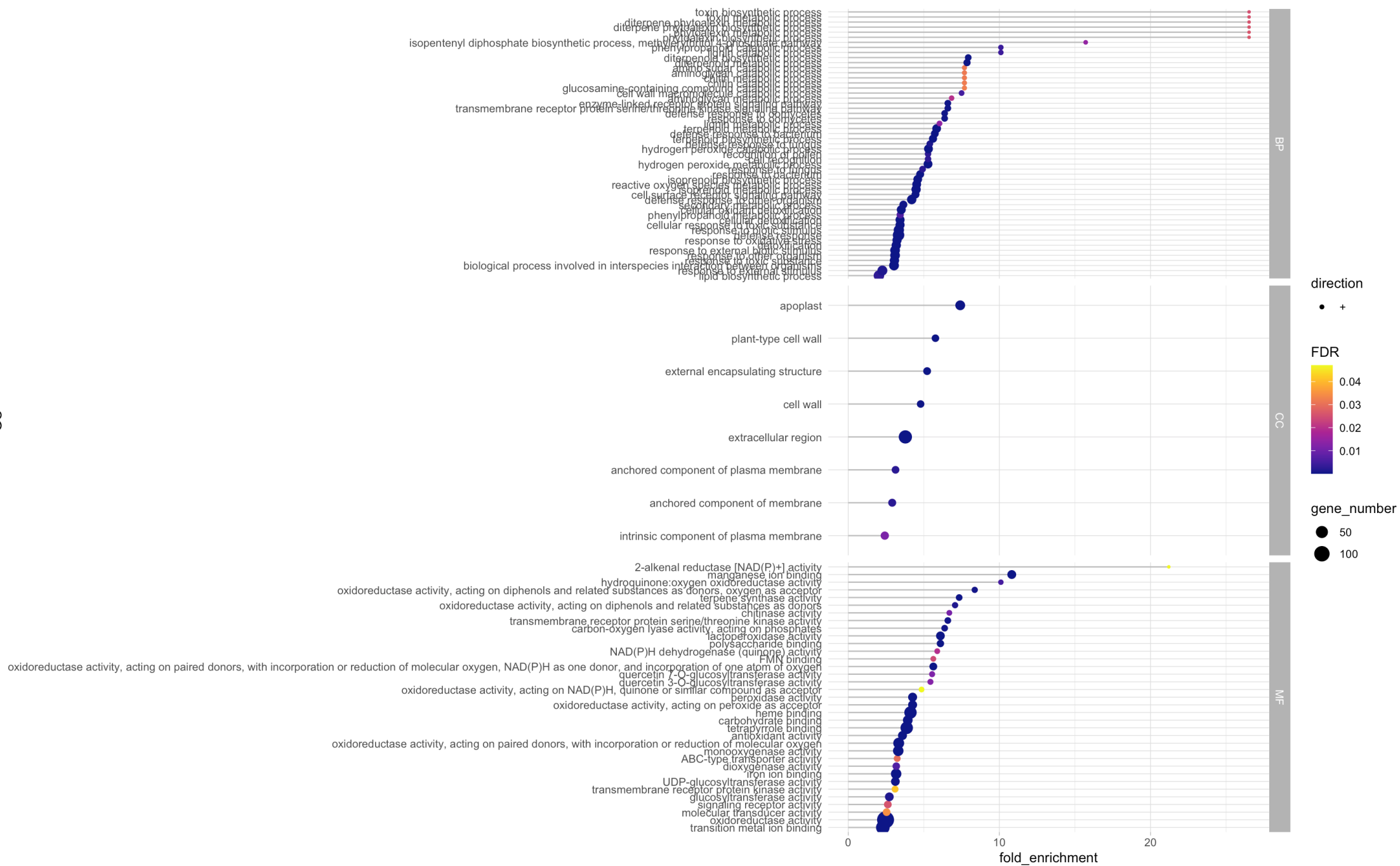

Supplement: Supplementary file 2 — Supplementary Material 2: Figures S4. Gene Ontology enrichment analysis of differentially expressed genes between treated and control samples within each MM line. [file 12870_2024_5697_MOESM2_ESM.pdf]
