# Supplementary material for: Transcriptome profiling of eight Zea mays lines identifies genes responsible for the resistance to Fusarium verticillioides
Source: BMC Plant Biol. 2024 Nov 21;24:1107. doi: 10.1186/s12870-024-05697-y (PMC11580207; doi:10.1186/s12870-024-05697-y)
Supplement: Supplementary file 3 — Supplementary Material 3: Methods S1. Contrasts description applied through edgeR for each biological question. [file 12870_2024_5697_MOESM3_ESM.pdf]

R

```
library(edgeR)

### make CONTRASTs to calculate DE within each line
##################################################
my.contrast <- makeContrasts(A632 = A632.treated - A632.control,
                             B73 = B73.treated - B73.control,
                             B96 = B96.treated - B96.control,
                             F7 = F7.treated - F7.control,
                             H99 = H99.treated - H99.control,
                             HP301 = HP301.treated - HP301.control,
                             Mo17 = Mo17.treated - Mo17.control,
                             W153R = W153R.treated - W153R.control,

                             TREATvsCONTR = (A632.treated + B73.treated + B96.treated + F7.treated + H99.treated + HP301.treated + Mo17.treated + W153R.treated)-
                               (A632.control + B73.control + B96.control + F7.control + H99.control + HP301.control + Mo17.control + W153R.control),

                             RESISTvsSUSCEP = ((A632.treated + H99.treated + HP301.treated + W153R.treated) - (A632.control + H99.control + HP301.control + W153R.control)) -
                               ((B73.treated + B96.treated + F7.treated + Mo17.treated) - (B73.control + B96.control + F7.control + Mo17.control)),

                             levels=design)



### BIO1 - conduct QL F-tests for the PATHOGEN EFFECT across lines
```

```
#################################################################
qlf <- glmQLFTest(fit, contrast = my.contrast[ , "TREATvsCONTR"])



### BIO2 - conduct QL F-tests for the PATHOGEN EFFECT on each LINE
#################################################################
### Significant differential expression in each gene using the QL F-test
A632 <- glmQLFTest(fit, contrast = my.contrast[,"A632"])
B73 <- glmQLFTest(fit, contrast = my.contrast[,"B73"])
B96 <- glmQLFTest(fit, contrast = my.contrast[,"B96"])
F7 <- glmQLFTest(fit, contrast = my.contrast[,"F7"])
H99 <- glmQLFTest(fit, contrast = my.contrast[,"H99"])
HP301 <- glmQLFTest(fit, contrast = my.contrast[,"HP301"])
Mo17 <- glmQLFTest(fit, contrast = my.contrast[,"Mo17"])
W153R <- glmQLFTest(fit, contrast = my.contrast[,"W153R"])


### BIOLOGICAL QUESTION 3 --> genes responsible for the RESISTANCE to the infection
### 4 most resistant vs 4 most susceptible (or vs 2 most susceptible)
###########################################################################################
### find genes that are differentially expressed resistant vs susceptible lines
qlf <- glmQLFTest(fit, contrast = my.contrast[ , "RESISTvsSUSCEP"])

```
